# Electricity and Medicine

**Published:** 1919-03-29

**Authors:** 


### ELECTRICITY AND MEDICINE.

To the large number of the younger generation of doctors who have in their war-time work included some first experiences of the application of electrical apparatus to medicine, the news that arrangements are in progress at several centres to develop post-graduate study of radiology and electrotherapeutics must be more than welcome. Some months ago, in view of the recent great advances in this work and the disadvantages which might result from the then frequent custom of unqualified practice in many complicated electrical manipulations, there was formed the British Association of Radiology and Electrotherapy, with Sir James Mackenzie Davidson as president. The committee elected not only decided to take steps leading to the provision of increased instruction by our medical schools in these highly technical subjects, but also made well-received representations that a diploma of efficiency should be granted at one of the leading universities.

Realisation is daily becoming more widespread that we have here to deal with a branch of medicine replete with possibilities and, considering the invaluable lessons of the war, worthy of immediate recognition and development. A first survey would suggest the predominant importance of $x$-ray diagnosis, but although this is the application of which the greater number of medical men have more or less knowledge, there are a variety of other electrical phenomena, with both high and low tension currents, which are turned to equally satisfactory use in both the treatment of disease and the elucidation of its mysteries. In home hospitals, before the war, it was too often the case that patients were sent to the "light department"—or whatever term was in local use—and there, except to the more ardent and inquisitive students, the treatment or investigations remained obscure, and were recorded only by some brief reference in the case report. Examining boards demanded but a relatively superficial knowledge of the subject, a mere word being sufficient to satisfy the examiner that the student knew when he should seek the electrical specialist's advice, and no real details of the relation between technical and clinical work were ever required. We are fast reaching a stage where team work and specialisation form the only truly efficient plan upon which any medical enquiry or research can be conducted, but one must remember that each unit of the team should have sufficient knowledge of other branches to provide him with power to appreciate and understand the work of his colleagues.

The details of medical fields touched upon by this fast developing science or even the mere enumeration of their main types is impossible in these few words, and one can only urge that the subject should receive the fullest recognition and that general instruction be given in its elements at least. In future we cannot afford to leave the electrical department's work out of the medical curriculum any more than we can omit bacteriology or public hygiene, and the efforts of the new association will be directed in an excellent cause.

